# Recording of ’COVID-19 vaccine declined‘: a cohort study on 57.9 million National Health Service patients’ records in situ using OpenSAFELY, England, 8 December 2020 to 25 May 2021

**DOI:** 10.2807/1560-7917.ES.2022.27.33.2100885

**Published:** 2022-08-18

**Authors:** Helen J Curtis, Peter Inglesby, Brian MacKenna, Richard Croker, William J Hulme, Christopher T Rentsch, Krishnan Bhaskaran, Rohini Mathur, Caroline E Morton, Sebastian CJ Bacon, Rebecca M Smith, David Evans, Amir Mehrkar, Laurie Tomlinson, Alex J Walker, Christopher Bates, George Hickman, Tom Ward, Jessica Morley, Jonathan Cockburn, Simon Davy, Elizabeth J Williamson, Rosalind M Eggo, John Parry, Frank Hester, Sam Harper, Shaun O’Hanlon, Alex Eavis, Richard Jarvis, Dima Avramov, Paul Griffiths, Aaron Fowles, Nasreen Parkes, Stephen JW Evans, Ian J Douglas, Liam Smeeth, Ben Goldacre

**Affiliations:** 1 The DataLab, Nuffield Department of Primary Care Health Sciences, University of Oxford, Oxford, United Kingdom; 2 London School of Hygiene and Tropical Medicine, London, United Kingdom; 3 TPP, TPP House, Leeds, United Kingdom; 4 EMIS Health, Leeds, United Kingdom

**Keywords:** COVID-19, SARS-CoV-2, vaccination, vaccine hesitancy, NHS England

## Abstract

**Background:**

Priority patients in England were offered COVID-19 vaccination by mid-April 2021. Codes in clinical record systems can denote the vaccine being declined.

**Aim:**

We describe records of COVID-19 vaccines being declined, according to clinical and demographic factors.

**Methods:**

With the approval of NHS England, we conducted a retrospective cohort study between 8 December 2020 and 25 May 2021 with primary care records for 57.9 million patients using OpenSAFELY, a secure health analytics platform. COVID-19 vaccination priority patients were those aged ≥ 50 years or ≥ 16 years clinically extremely vulnerable (CEV) or ’at risk’. We describe the proportion recorded as declining vaccination for each group and stratified by clinical and demographic subgroups, subsequent vaccination and distribution of clinical code usage across general practices.

**Results:**

Of 24.5 million priority patients, 663,033 (2.7%) had a decline recorded, while 2,155,076 (8.8%) had neither a vaccine nor decline recorded. Those recorded as declining, who were subsequently vaccinated (n = 125,587; 18.9%) were overrepresented in the South Asian population (32.3% vs 22.8% for other ethnicities aged ≥ 65 years). The proportion of declining unvaccinated patients was highest in CEV (3.3%), varied strongly with ethnicity (black 15.3%, South Asian 5.6%, white 1.5% for ≥ 80 years) and correlated positively with increasing deprivation.

**Conclusions:**

Clinical codes indicative of COVID-19 vaccinations being declined are commonly used in England, but substantially more common among black and South Asian people, and in more deprived areas. Qualitative research is needed to determine typical reasons for recorded declines, including to what extent they reflect patients actively declining.

## Introduction

On 8 December 2020, the National Health Service (NHS) in England administered the first coronavirus disease (COVID-19) vaccination as part of a vaccine campaign to combat the ongoing COVID-19 pandemic. General practices were required to ensure that in addition to any national call/re-call service, they used existing tools to write, text or call patients [1]. By mid-April 2021, all people in England included in the initial Joint Committee on Vaccination and Immunisation (JCVI) vaccine priority groups (Table 1) had been invited to receive a COVID-19 vaccination. Following this, invitations were extended to all other adults [2]. We have previously described detailed trends and clinical characteristics of COVID-19 vaccine recipients using 57.9 million patients’ records [3] and have published a weekly report (https://reports.opensafely.org/reports/vaccine-coverage). During the campaign, 19 of 20 people aged ≥ 50 years received their first vaccination. However, concerns remained around lower vaccine coverage in some groups, particularly ethnic minorities [3,4].

**Table 1:** Priority groups for COVID-19 vaccination advised by the Joint Committee on Vaccination and Immunisation (JCVI) [8], compared with the priority groups used in this report, England

Priority group	JCVI risk group [8]	Groups used in this report		
		Priority group	Group name	Combined group
1	Residents in a care home for older adults Staff working in care homes for older adults	Residents in a care home for older adults, aged ≥ 65 years	Care home	≥ 65
2	All those aged ≥ 80 years Frontline Health and social care workers	All those aged ≥ 80 years	≥ 80	
3	All those aged ≥ 75 years of age	All those aged ≥ 70 years	70–79	
4	All those aged ≥70 years and clinically extremely vulnerable individuals (not including pregnant women and those aged < 16 years)	Clinically extremely vulnerable individuals (not including pregnant women and those aged < 16 years)	CEV	CEV/at risk
5	All those aged ≥ 65 years		65–69	≥ 65
6	Adults aged 16–65 years in an at risk group		At risk	CEV/at risk
7	All those aged ≥ 60 years		60–64	50–64
8	All those aged ≥ 55 years		55–59	
9	All those aged ≥ 50 years		50–54	

CEV: clinically extremely vulnerable; JCVI: Joint Committee on Vaccination and Immunisation.

The final column indicates how priority groups are combined into three larger groups where this was necessary for data presentation. Although pregnant women were not included in the CEV or at risk groups on the basis of their pregnancy, some pregnant women will be included in these groups based on other criteria. Each patient was assigned only to their highest priority group and not included again as part of any other priority group.

In England, electronic health record (EHR) software has the functionality to record when a vaccination has been declined, and Systematized Nomenclature of Medicine -- Clinical Terms (SNOMED-CT), the mandated coding language in NHS primary care, has several codes that may be used for this purpose (Supplementary Table S2A lists the SNOMED codes related to COVID-19 vaccines being declined). These codes may be used where a patient has explicitly and absolutely refused a vaccine; however, they may also sometimes be used for other reasons, such as when a patient wishes to delay getting the vaccine, e.g. because of illness, or rejects a vaccine invitation from one organisation after booking an appointment to be vaccinated elsewhere. An additional range of codes are available to indicate other situations including contraindications, vaccination appointments being missed or the vaccine being ‘not given’ (Supplementary Table S2B lists the SNOMED codes related to COVID-19 vaccinations not being done), but their usage may occasionally cross over. As there is no comprehensive national guidance or specification on how general practices should use these codes, the individual general practices may also use them to facilitate the organisational delivery of this large-scale vaccination campaign. For example, in order to prevent further automated invitations from the EHR system, a general practice may add a code indicating a patient has declined when no response has been received after a certain number of invitations, or they may be used in uncertain circumstances such as to note a possible intolerance.

In this study, we aimed to describe the patterns in recorded COVID-19 vaccine declines among 24.5 million priority patients, by examining the pseudonymised records of 57.9 million patients (ca 95% of registered patients in England) held in the OpenSAFELY platform, a secure analytics platform for NHS patient data [5].

## Methods

### Study design

We conducted a retrospective cohort study using general practice primary care EHR data from all England general practices supplied by the EHR software providers EMIS and TPP (ca 95% of registered patients in England). Follow-up began on 8 December 2020, the start of the national vaccination campaign, and ended on 25 May 2021, which was the latest date available at the time of analysis and more than 1 month after all those in priority groups had been offered a vaccination [2].

### Study population

We included all patients registered with a general practice in England using EMIS or TPP software on 25 May 2021 and identified as belonging to a vaccine priority group (Table 1). We additionally excluded patients with an unknown date of birth (i.e. age > 120 years) or sex.

### Data source

Primary care records managed by EMIS and TPP were accessed through OpenSAFELY, an open-source data analytics platform created by our team on behalf of NHS England to address urgent COVID-19 research questions (https://opensafely.org). OpenSAFELY provides a secure software interface allowing a federated analysis of pseudonymised primary care patient records from England in near real-time within the EMIS and TPP highly secure data environments. Non-disclosive, aggregated results are exported to GitHub (https://github.com) where further data processing and analysis takes place. This avoids the need for large volumes of potentially disclosive pseudonymised patient data to be transferred off-site. This, in addition to other technical and organisational controls, minimises any risk of re-identification. The dataset available to the platform includes pseudonymised data such as coded diagnoses, medications and physiological parameters. No free text data are included. All activity on the platform is publicly logged and all analytic code as well as supporting clinical coding lists are automatically published. In addition, the framework provides assurance that the analysis is reproducible and reusable. Further details on our information governance, ethics and platform can be found below in the Ethical Statement at the end of the article.

### COVID-19 vaccine status

Vaccine administration details are recorded in the National Immunisation Management Service (NIMS) and electronically transmitted to every individual’s general practitioner (GP) record on a daily basis. We ascertained which patients had any recorded COVID-19 vaccine administration code in their primary care record. We also captured other clinical codes for a COVID-19 vaccination that may have been entered outside of the usual system (Supplementary Table S1 lists the SNOMED codes related to COVID-19 vaccines being given). Patients were considered to be vaccinated if any COVID-19 vaccination record or code was present, irrespective of the number of doses received.

In March 2021, NHS Digital published a news article listing COVID-19 vaccination codes [6]. From this document, we identified all codes indicative of declining a COVID-19 vaccine as those containing the word ‘declined’ in the description (Supplementary Table S2A). We included three additional codes fitting this pattern, either reported in the national COVID-19 Vaccination Uptake Reporting Specification [7], or inactive codes. Patients were assigned to the declined group if they had any code for a COVID-19 vaccination being declined, irrespective of their vaccination status. We describe subsets of the declined group as follows: (i) those who had already had a COVID-19 vaccination (declined post-vaccination), (ii) those who later received their first dose, at minimum a day after the decline was recorded (declined then received) and (iii) those with no recorded vaccination (remaining declined). In patients with no recorded vaccination or declined code, we looked for any other records indicating an attempt or intention to be vaccinated, e.g. a contraindication or ‘did not attend’ (Supplementary Table S2B) and assigned these patients to the ’contraindicated/unsuccessful’ group. All other unvaccinated patients were assigned to the ’no records’ group.

### Priority groups for vaccination

We classified patients into nine priority groups (Table 1) using SNOMED-CT codelists and logic defined in the national COVID-19 Vaccination Uptake Reporting Specification developed by PRIMIS v1.1 [7]. These nine groups were targeted for vaccination in the first phase of the roll-out in England (December 2020–April 2021), and included everyone aged 50 years and older, health and care workers and those aged 16 years and older at increased clinical risk from COVID-19. There were two groups at increased risk, the clinically extremely vulnerable (CEV) who were advised to ’shield’ to reduce the risk of infection, and those at risk, who did not receive that recommendation [8]. However, in order to report age groups and clinical groups separately, we combine the cohorts aged 70–74 and 75–79 years together as Group 3, leaving the CEV cohort separately as Group 4. We also limited the care home population to those aged 65 years and older (Table 1). We did not assess eligibility for the relevant priority groups in our analysis as defined by occupation, i.e. health and care workers [2,3] because this information was largely missing or unreliable in GP records. These patients were classified into a lower priority group where applicable (e.g. by clinical risk or age) and were otherwise excluded. Each patient was assigned only to their highest priority group and not included again as part of any other priority group. In line with the national reporting specification, age was calculated as on 31 March 2021, while other criteria were ascertained using the latest available data at the time of analysis.

### Key demographic and clinical characteristics

We extracted all patient demographics defined by the national reporting specification, e.g. ethnicity. We made a small modification to the definition of pregnancy, restricting this to females aged under 50 years, to avoid including any codes incorrectly recorded for males and post-menopausal women. We also extracted demographics not defined by the specification, including the Index of Multiple Deprivation (IMD; 2019 values [9]), derived from patient postcodes at Lower Layer Super Output Area level [10], grouped into quintiles.

### Codelists and implementation

Information on all characteristics were obtained from electronic primary care records by searching TPP SystmOne and EMIS records for specific coded data. EMIS (https://www.emishealth.com) and TPP SystmOne (https://tpp-uk.com) are fully compliant with the mandated NHS standard of SNOMED-CT clinical terminology. Medicines are entered or prescribed in a format compliant with the NHS Dictionary of Medicines and Devices (dm + d) [11]. Codelists and logic for most features in the national reporting specification were automatically converted to OpenSAFELY software. 

### Missing data

Patients with missing ethnicity or IMD information are included as ‘unknown’. A very small number of patients’ vaccinations (0.0012%) or declines (0.051%) were dated before the start of the vaccination campaign or lacked a date altogether. Accuracy was prioritised in determining whether a decline was recorded before a vaccination so these patients for whom the precise sequence could not be determined were counted in the ‘declined post-vaccination’ group. Codes specifically relating to vaccine allergy or contraindications could not be retrieved from the EMIS system, so a small number of affected patients will be counted in the unvaccinated ‘no records’ group.

### Study measures

We calculated the daily cumulative number and rate of COVID-19 vaccinations, coded vaccine declines, those with contraindications/unsuccessful vaccinations and those with no records related to vaccination, for each priority group. We also measured how many people were vaccinated after previously being recorded to have declined, and the time between these records (0 to < 2 weeks, 2 to < 4 weeks, 1 to < 2 months, ≥ 2 months), presented as time trends, bar charts and brief descriptive statistics. We assessed the rate of declines recorded at practice level per thousand patients, excluding practices with 250 or fewer registered patients in priority groups and those with less than 10 vaccinated patients, and presented this as a histogram and heatmap. Patient counts were rounded to the nearest 7 and values under 7 suppressed before release from each EHR system; practice counts of 1–3 were shown as 2.

### Software and reproducibility

Data management and analysis was performed using the OpenSAFELY software libraries (https://www.opensafely.org) and Python (https://www.python.org), both implemented using Python 3. This analysis was delivered using federated analysis through the OpenSAFELY platform. Codelists and code for data management and data analysis were specified once using the OpenSAFELY tools. These were then transmitted securely to the OpenSAFELY-TPP platform within TPP’s secure environment, and separately to the OpenSAFELY-EMIS platform within EMIS’s secure environment, where they were each executed separately against local patient data. Summary results were then reviewed for disclosiveness, released and combined for the final outputs. 

Patients were not formally involved in developing this specific exploratory study that was produced rapidly in the context of the rapid vaccine rollout during a global health emergency. We have developed a publicly available website https://opensafely.org through which we invite any patient or member of the public to contact us regarding this study or the broader OpenSAFELY project.

## Results

We present patient counts rounded to the nearest 7 to reduce identifiable patient information. Of 57.9 million patients in total, 24.5 million (42.3%) were identified as being in priority groups, of whom 21.8 million (89.2%) had received at least one COVID-19 vaccine by 25 May 2021 (Table 2). Some 663,033 (2.7%) were recorded with a code suggestive of declining a vaccine, 125,587 (18.9%) of whom were later vaccinated, while 53,543 (8.1%) had already had at least one vaccination (Table 2). Thus, 483,791 (2.0%) people in priority groups have been recorded as declining and remain unvaccinated. Only 15,015 patients (0.1%) had no vaccine or decline recorded but had a recorded contraindication or unsuccessful vaccination, e.g. did not attend an appointment, while an additional 2,155,076 (8.8%) had no records of vaccination, decline, contraindication or other vaccine-related codes.

**Table 2:** Summary of COVID-19 vaccination status and declines recorded for patients in OpenSAFELY by priority group, England, 8 December 2020–25 May 2021 (n = 24,476,809)

Group	Total population	Vaccinated		Declined								Contra- indicated/ unsuccessful		No records	
				Total declined		Declined then received		Declined post- vaccine		Remaining declined					
		n	%	n	%	n	%	n	%	n	%	n	%	n	%
Care home	252,637	242,697	96.1	6,335	2.5	1,617	0.6	714	0.3	3,990	1.6	63	0.0	5,887	2.3
≥ 80	2,578,870	2,467,794	95.7	86,520	3.4	26,530	1.0	6,230	0.2	53,746	2.1	966	0.0	56,364	2.2
70–79	4,754,792	4,530,071	95.3	128,310	2.7	27,321	0.6	16,667	0.4	84,315	1.8	2,450	0.1	137,956	2.9
CEV	1,991,549	1,733,942	87.1	87,066	4.4	16,695	0.8	5,404	0.3	64,946	3.3	1,904	0.1	190,757	9.6
65–69	2,503,298	2,311,820	92.4	58,275	2.3	8,337	0.3	4,228	0.2	45,703	1.8	1,449	0.1	144,326	5.8
At risk	4,324,663	3,592,463	83.1	128,989	3.0	16,877	0.4	6,188	0.1	105,910	2.4	3,479	0.1	622,811	14.4
60–64	2,152,038	1,912,043	88.8	44,352	2.1	6,594	0.3	4,130	0.2	33,614	1.6	1,253	0.1	205,128	9.5
55–59	2,804,333	2,426,235	86.5	60,858	2.2	12,320	0.4	6,062	0.2	42,462	1.5	1,603	0.1	334,033	11.9
50–54	3,114,629	2,605,862	83.7	62,328	2.0	9,296	0.3	3,920	0.1	49,105	1.6	1,848	0.1	457,814	14.7
**All**	**24,476,809**	**21,822,927**	**89.2**	**663,033**	**2.7**	**125,587**	**0.5**	**53,543**	**0.2**	**483,791**	**2.0**	**15,015**	**0.1**	**2,155,076**	**8.8**

CEV: clinically extremely vulnerable; COVID-19: coronavirus disease.

‘Vaccinated’ includes patients who were previously recorded to have declined (these are also shown separately in the ‘Declined then received’ column). ‘Contraindicated/unsuccessful’ includes patients with no recorded vaccination or declined code, but with any other code indicative of contraindication or an unsuccessful attempt to vaccinate, e.g. ‘did not attend’ (Supplementary Table S1 lists the SNOMED codes related to COVID-19 vaccines being given). ‘Total population’ represents the sum of ‘Vaccinated’ + ’Remaining declined’ + ’Contraindicated/unsuccessful’ + ’No records’. Patient counts are rounded to the nearest 7 to reduce the risk of disclosure of identifiable patient information, i.e. 13 patients were reported as 14, 25 patients as 21 etc.

### Individual priority groups

**Figure 1:**
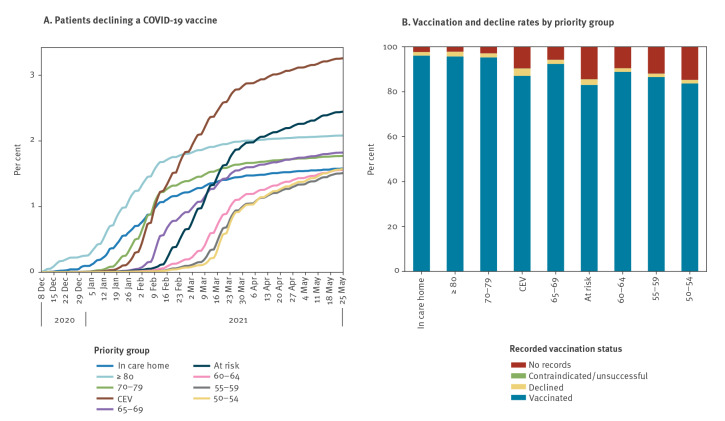
Recorded COVID-19 vaccination status of patients in OpenSAFELY, England, 8 December 2020–25 May 2021 (n = 24,476,809)

**Figure 2:**
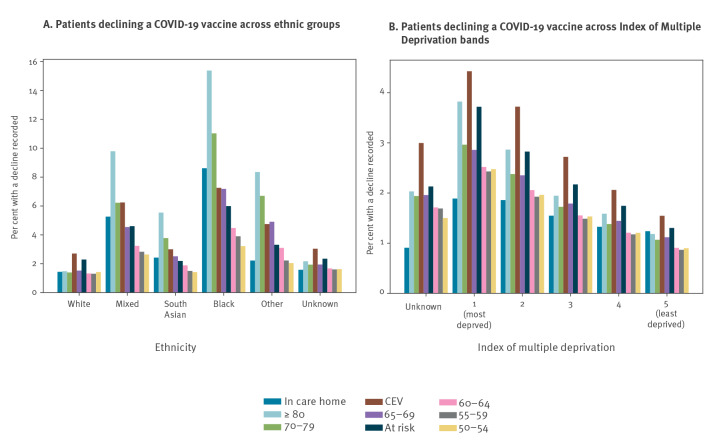
Percentage of patients who had a COVID-19 vaccination decline recorded and were unvaccinated in OpenSAFELY by priority group, England, as per 25 May 2021 (n = 483,791)

IMD: index of multiple deprivation; COVID-19: coronavirus disease.

The total rate of declines being recorded was highest in the CEV group at 4.4%, followed by ≥ 80 (3.4%), and at risk (3.0%) (Table 2). Removing those who were vaccinated, CEV remained the highest with 3.3% recorded as declining (−1.1% absolute reduction), with those ≥ 80 reducing to 2.1% (−1.3% absolute reduction), with a smaller reduction for the at risk group to 2.4% (–0.6% absolute reduction) (Figures 1 and 2; see Supplementary Table S1 for the SNOMED codes). Within the CEV/at risk groups there was a strong correlation with age group, and comparing each 5-year age band with other priority groups, the percentage of people recorded as declining was still higher in the CEV/at risk group, e.g. 1.9% vs 1.6% for 60–64 (see Supplementary Table S3 for the vaccination status in each combined priority group); however, the percentage vaccinated in each age band was also higher. Patients recorded as declining a COVID-19 vaccination accounted for approximately half of those currently unvaccinated among the ≥ 80 group, and more than one third in the other three top eligibility groups (care home, 70–79 and CEV; Figure 1 and Table 2).

COVID-19: coronavirus disease.

A. Cumulative percentage of patients in each priority group recorded as declining a COVID-19 vaccination and remaining unvaccinated.

B. Recorded COVID-19 vaccination status for patients by priority group. 'Declined’ excludes patients with a recorded vaccination.

‘Vaccinated’ includes those previously recorded as declining.

### Variation by demographic factors

**Figure 3:**
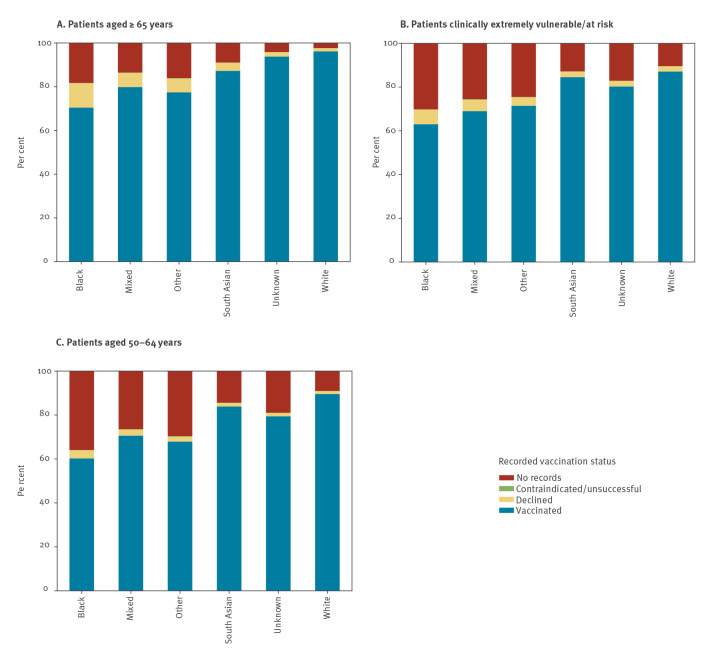
COVID-19 vaccination status recorded for patients in OpenSAFELY for three combined priority groups split by broad ethnic groups, England, as per 25 May 2021 (n = 24,476,809)

The percentage of patients in each ethnic group who had a decline recorded and were unvaccinated, split by individual priority group, is shown in Figure 2A (time trends are presented in Supplementary Figure S1 by combined priority groups (aged ≥ 65 years, CEV/at risk, aged 50–64 years)). The percentage of the white population who were recorded as declining (and unvaccinated) was similar across each priority group (1.3–1.5%), except for CEV and at risk groups which were slightly higher at 2.7% and 2.3%, respectively (Figure 2A). The variation in most other ethnic groups was more marked, especially in the black population. The highest rate within the black population was 15.3% (aged ≥ 80 years), more than 10 times greater than the white population aged ≥ 80 years (1.5%), while the lowest rate was 3.2% (aged 50–54 years), still higher than any of the groups in the white population. The percentage recorded as declining (and unvaccinated) in the South Asian population was generally lower than other non-white groups, ranging from 1.4% (aged 50–54) to 5.6% (aged ≥ 80 years) (Figure 2A). Time trend charts show that these differences have been consistent but increasing over time (see Supplementary Figure S1A-C for the cumulative percentage of patients recorded as declining a COVID-19 vaccination). There was also a much larger proportion of people in each ethnic minority group with no records of vaccination with no reason recorded compared with the white population (Figure 3).

COVID-19: coronavirus disease.

‘Declined’ excludes patients with a recorded vaccination. ‘Vaccinated’ includes those previously recorded as declining.

There was a clear trend towards increased recording of vaccination declines with increasing deprivation across priority groups (least deprived quintile: 0.9–1.5%, most deprived: 2.4–4.4% excluding care homes; Figure 2B). This divergence by deprivation level was also consistently increasing over time (Supplementary Figure S1D-F provides further detail on the rate of vaccination declined by deprivation group). Presence of a severe mental health condition was associated with lower vaccination rates and more declines being recorded, and a similar but less divergent pattern was seen in those with a learning disability (Supplementary Table S3A and B shows the vaccine status in combined priority groups by demographic features). Among all those with a recent pregnancy (only applicable in the CEV/at risk group), vaccination rates were much lower compared with others of childbearing age (38.0% in CEV/at risk vs 67.1% and 72.6% for groups aged 16–29 and 30–39 years, respectively), more declines were recorded (5.9% vs 4.2% and 3.7%), and more had no vaccine-related records (55.7% vs 28.6% and 23.6% (Supplementary Table S3B).

### Patients who were recorded as declining and later had a COVID-19 vaccination

**Figure 4:**
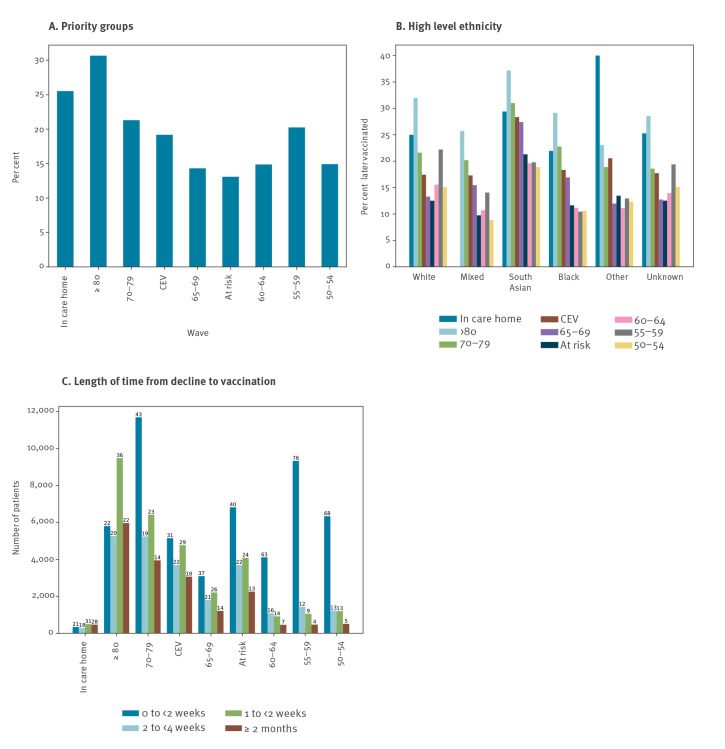
Patients in each priority group who were later vaccinated, after previously being recorded as declining a COVID-19 vaccine, England, as per 25 May 2021 (n = 125,587)

Of all those in priority groups who have had a decline recorded at any point, 18.9% later received a vaccination (Table 2). This conversion rate from ‘declined’ to ‘vaccinated’ ranged from 13.1% in the at risk group to 30.7% in the group aged ≥ 80 years (Figure 4A). This pattern was broadly similar in each ethnic group, but was generally higher in the South Asian population, which had the highest conversion rate in all but two priority groups (Figure 4B). Among combined cohorts aged ≥ 65 years, CEV/at risk, and aged 50–64 years, the conversion rates in the South Asian population were 32.3%, 25.2%, and 19.3%, respectively, vs 22.8%, 15.5%, and 16.8% for all other ethnicities combined. The time delay between the recorded decline and the first dose being received was primarily 0–2 weeks in the group aged 50–64 years in contrast to the older groups (≥ 65 years) which had a wider range of time delays.

COVID-19: coronavirus disease.

A. Percentage of those in each priority group who were later vaccinated.

B. Percentage of those in each priority group who were later vaccinated by ethnicity and priority group.

C. Number of patients in each priority group who were later vaccinated, by length of time between the recorded decline and the vaccination (labels show percentages). The youngest eligible group (aged 50–54 years) was eligible for only 1 month at the time of analysis, although some healthcare workers counted in this group would have been eligible much longer.

### Variation by general practice

**Figure 5:**
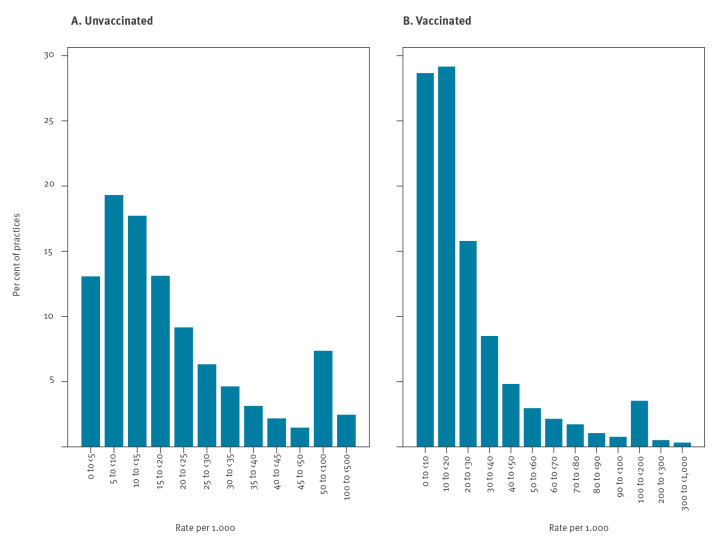
Histograms showing variation in the number of patients in priority groups per practice recorded as declining a COVID-19 vaccination, England, as per 25 May 2021 (n = 663,033)

Almost all practices (6,290/6,364; 98.8%) had at least one patient recorded as declining the vaccine (limited to practices with > 250 patients in priority groups and > 10 vaccinated patients). There was a broad range of rates per practice, with just over half of practices having fewer than 15 patients recorded as declining per 1,000 registered patients, and most (90%) having 50 or fewer recording a decline (Figure 5A). The majority of practices (90%) had 60 or fewer declines recorded per 1,000 vaccinated patients (Figure 5B). However, there was a long tail with some practices having 300 or more recorded declines per 1,000 vaccinated patients. Plotting against the number of priority group patients per practice indicates no strong correlation with practice size, although smaller practices were slightly more likely to have higher rates of declines recorded (Supplementary Figure S2 provides a heatmap of practice level rates of declined COVID-19 vaccination).

Only includes unvaccinated patients. Practices with 250 or fewer registered patients in priority groups and those with 10 or fewer vaccinated patients were excluded.

## Discussion

Overall, of currently registered people in priority groups in England, almost half a million (2.0%) have been recorded as declining a COVID-19 vaccine and remained unvaccinated as of 25 May 2021, while 8.8% were unvaccinated without a recorded reason. Other reasons for lack of vaccination, such as contraindications, were rarely recorded (0.1%). Recorded declines were most common in the CEV group. Patients from ethnic minority groups and more deprived areas had higher rates of vaccine decline codes. Codes for declining COVID-19 vaccines were present in almost all practices, but there was substantial variation in rates. Of all those in priority groups who had a decline recorded at any point, 18.9% were later vaccinated.

Among priority groups, the proportion of people recorded as declining and being unvaccinated was highest in the CEV and at risk groups, even when comparing individual 5-year age bands. In a 2020 survey, CEV, i.e. shielding, status was associated with lower self-reported COVID-19 vaccine hesitancy [12]. However, we also found the percentage who were vaccinated was higher in the CEV/at risk groups compared with others of the same age. Therefore, a likely explanation for our finding is that those at increased risk because of their clinical conditions were sent more follow-up invitations, giving a greater opportunity for a vaccine to be administered or a decline to be recorded.

Our finding of higher rates of declines being recorded in black and South Asian groups is generally consistent with survey data on intention to accept the COVID-19 vaccine [13-17], and with previous research on variation in vaccine coverage in other vaccination campaigns historically [18-20]. Similar patterns have also been observed in other European countries and the United States (US) [21,22].

We found that 18.9% of those initially recorded as declining were later vaccinated. As well as those who genuinely changed their mind, this will include some patients who were initially undecided, temporarily declined (e.g. due to illness), rejected a repeated invitation after already booking a vaccine or had a decline recorded in error or for administrative reasons. It may also reflect changing preferences over time. A survey in April 2021 indicated that 52% of those reporting they would definitely not have the vaccine in November/December 2020 later accepted it when offered (and 15% of those not yet offered were likely to accept) [15], while another survey noted a reduction in hesitancy from 26.9% in October 2020 to 16.9% in January/February 2021 [23], indicating substantial shifts in preferences as the campaign has progressed. In the US, a survey revealed a high prevalence of some degree of hesitancy even among those being vaccinated (60%), with levels varying by age and ethnicity [24]. We found the probability of going from recorded as declining to being vaccinated was broadly similar across ethnic groups, but slightly higher in the South Asian population. Qualitative research could validate this finding, for example by surveying a subset of patients declining vaccination to verify that their initial intentions were reflected in their records.

The key strength of this study is its unprecedented scale: our source population includes 57.9 million people, over 95% of the population in England. Another key strength is that we identified patients in JCVI priority groups by directly implementing the full official SNOMED-CT codelists and logic for the national specification, thus ensuring that our cohorts are perfectly in line with national procedures and clinician expectations.

The rate of declines recorded was likely influenced by the length of time each group was eligible for vaccination, and the number of times practices attempted to contact them, highlighting the need for ongoing monitoring. For example, the group aged ≥ 80 years were invited from the start of the campaign (December 2020), while the group aged 50–54 were invited from mid-March 2021 [25]. The data flow from mass vaccination centres is thought to be largely complete, but some vaccination records may be missing, e.g. vaccines delivered in inpatient settings; this may disproportionately affect the CEV and at risk groups, for example those with kidney disease [26], as such groups are more likely to have inpatient hospital stays. It is possible that some patients had not been approached at all, although all priority group patients had reportedly been offered the vaccine over 1 month before our latest data update [2]. However, some patients with no vaccine-related records may not have been reached, e.g. outdated/incorrect contact details or have left the country. They may alternatively have chosen to decline by not responding to the invitations, or were undecided. Patients who did not attend are likely to be vastly underrepresented here as data from mass vaccination centres only include successful vaccinations, therefore patients who did not attend will only be recorded for appointments booked in practice settings.

Almost all practices had at least one patient with a declined code, indicating that these codes were widely used. However, there was substantial variation between practices. With no national guidance on the use of decline codes it is likely that there was variation in use, as well as variation in patient preference in different localities. For example, some practices may have applied them to patients who simply did not respond after several contact attempts. As such, we recommend a detailed survey and/or qualitative research with patients and NHS staff to provide more descriptive information on how these codes are being used, and to what extent they reflect patient intentions, e.g. whether patients with decline codes were typically more strongly hesitant than those with no records, and also to shed light on any reasons for differences between groups. It would also be useful to investigate how similar codes are used in other routine vaccination campaigns.

Our findings may have implications for targeting patients for vaccinations in future, e.g. using targeted communication approaches dependent on the presence or absence of a decline. It has been shown that certain messaging can influence those who are most hesitant about COVID-19 vaccination [23]. By using electronic health records for decline codes, such interventions could be targeted and automated on a large scale.

We also recognise some limitations. Our population, though extremely large, may not be fully representative: it does not include individuals not registered with a general practice, or the 4% of patients registered at practices not using TPP or EMIS. We included only currently registered and living patients, and excluded those who have moved away or died during the vaccination campaign. Primary care records cannot reliably be used to determine vaccine eligibility through reasons of employment, and as such our priority groups which include working-age people will contain a subset who were offered the vaccination earlier than others. The analysis of variation in declining vaccination among different ethnicity groups was restricted to broad groupings in this initial exploratory analysis; exploration of variation using a finer categorisation of ethnicities is an area for further development.

## Conclusions

Clinical codes indicative of COVID-19 vaccinations being declined are widely recorded in English general practice following the initial COVID-19 vaccination campaign. Vaccination declines were more common in patients from deprived areas and black and South Asian populations. The reasons for this require further exploration, and we suggest questionnaires and qualitative work, including among those who ultimately decide to receive a vaccine.

## Supplementary Data

1708000 Supplementary Material
