# Supplementary material for: Recording of ’COVID-19 vaccine declined‘: a cohort study on 57.9 million National Health Service patients’ records in situ using OpenSAFELY, England, 8 December 2020 to 25 May 2021
Source: Euro Surveill. 2022 Aug 18;27(33):2100885. doi: 10.2807/1560-7917.ES.2022.27.33.2100885 (PMC9389857; doi:10.2807/1560-7917.ES.2022.27.33.2100885)

This supplementary material is hosted by *Eurosurveillance* as supporting information alongside the article *Recording of "COVID-19 vaccine declined" in England: a cohort study on 57.9 million NHS patients' records in situ using OpenSAFELY*, on behalf of the authors, who remain responsible for the accuracy and appropriateness of the content. The same standards for ethics, copyright, attributions and permissions as for the article apply. Supplements are not edited by *Eurosurveillance* and the journal is not responsible for the maintenance of any links or email addresses provided therein.

Table S1. Classification of SNOMED codes related to COVID-19 vaccines being given. *These clinical codes will reflect only a small minority of cases, as vaccines are almost always recorded according to the product given.*

SNOMED Code	Description	Codelist
90640007	Coronavirus vaccination	COV-19 vacc given
840534001	Severe acute respiratory syndrome coronavirus 2 vaccination	covadm1
1324671000000103	Immunisation course to achieve immunity against SARS-CoV-2 (severe acute respiratory syndrome coronavirus 2)	COV-19 Vacc given
1324681000000101	Administration of first dose of SARS-CoV-2 (severe acute respiratory syndrome coronavirus 2) vaccine	Covadm1<br>COV-19 vacc given
1324691000000104	Administration of second dose of SARS-CoV-2 (severe acute respiratory syndrome coronavirus 2) vaccine	Covadm2<br>COV-19 vacc given
1324851000000106	SARS-CoV-2 (severe acute respiratory syndrome coronavirus 2) immunisation course started	COV-19 vacc given

Sources: COVID-19 Vaccination Codes *(NHS Digital 2021); National COVID-19 Vaccination Uptake Reporting Specification ("COVID-19 Vaccination Uptake Reporting Specification" n.d.)*

Table S2. Classification of SNOMED codes related to COVID-19 vaccines being (a) declined or (b) otherwise not done. *"Not done" codes are only counted for patients with no record of any dose being given.*

Classifi-cation	SNOMED Code	Description	Codelist(s)
**(a) Declined**	1324741000000101	SARS-CoV-2 (severe acute respiratory syndrome coronavirus 2) vaccination first dose declined	1st dose declined
	1324811000000107	SARS-CoV-2 (severe acute respiratory syndrome coronavirus 2) immunisation course declined	1st dose declined<br>2nd dose declined
	1324721000000108	SARS-CoV-2 (severe acute respiratory syndrome coronavirus 2) vaccination dose declined	1st dose declined<br>2nd dose declined
	1240651000000109	*[inactive]* SARS-CoV-2 (severe acute respiratory	1st dose declined

		syndrome coronavirus 2) vaccination declined	2nd dose declined
	1324751000000103	SARS-CoV-2 (severe acute respiratory syndrome coronavirus 2) vaccination second dose declined	2nd dose declined
**(b) Not done**	1240631000000102	Did not attend SARS-CoV-2 vaccination	1st appt DNA<br>2nd appt DNA
	1324831000000104	Did not attend for first dose of SARS-CoV-2 (severe acute respiratory syndrome coronavirus 2) vaccine	1st appt DNA
	1324841000000108	Did not attend for second dose of SARS-CoV-2 (severe acute respiratory syndrome coronavirus 2) vaccine	2nd appt DNA
	1324661000000105	Adverse reaction to SARS-CoV-2 (severe acute respiratory syndrome coronavirus 2) vaccine	allergy/contra
	1324711000000102	Allergy to SARS-CoV-2 (severe acute respiratory syndrome coronavirus 2) vaccine	allergy/contra
	1324731000000105	SARS-CoV-2 (severe acute respiratory syndrome coronavirus 2) immunisation course not indicated	allergy/contra
	1324761000000100	SARS-CoV-2 (severe acute respiratory syndrome coronavirus 2) immunisation course contraindicated	allergy/contra
	1240661000000107	*[inactive]* SARS-CoV-2 (severe acute respiratory syndrome coronavirus 2) vaccination contraindicated	allergy/contra
	1240671000000100	*[inactive]* SARS-CoV-2 (severe acute respiratory syndrome coronavirus 2) vaccination not indicated	allergy/contra
	1324821000000101	SARS-CoV-2 (severe acute respiratory syndrome coronavirus 2) immunisation course not done	1st dose not given
	1240681000000103	*[inactive]* Severe acute respiratory syndrome coronavirus 2 vaccination not done	1st dose not given
	1324771000000107	SARS-CoV-2 (severe acute respiratory syndrome coronavirus 2) vaccination dose not given	1st dose not given
	1324781000000109	SARS-CoV-2 (severe acute respiratory syndrome coronavirus 2) vaccination first dose not given	1st dose not given
	1324791000000106	SARS-CoV-2 (severe acute respiratory syndrome coronavirus 2) vaccination second dose not given	2nd dose not given
	1324861000000109	SARS-CoV-2 (severe acute respiratory syndrome coronavirus 2) immunisation course abandoned	2nd dose not given
	1240701000000101	Severe acute respiratory syndrome coronavirus 2 vaccine not available	COV19 vacc unavailable

Source:* *COVID-19 Vaccination Codes *(NHS Digital 2021); plus three additional codes were identified in the national COVID-19 Vaccination Uptake Reporting Specification ("COVID-19 Vaccination Uptake Reporting Specification" n.d.), or in a SNOMED browser. Inactive codes indicate those which are no longer available for use but may have been entered in patient records previously.*

Table S3

Percentage of population in each combined priority group [(a) 65+ (b) CEV/At Risk, (c) 50-64] who are recorded as vaccinated, declined and unvaccinated, contraindicated/unsuccessful, or with no vaccine records, according to demographic features, as at 25 May 2021. *Percentages may not sum to 100 due to rounding. Patient counts rounded to the nearest 7.*

Table 3a. Ages 65+ (including care home residents)

Category	Group	Total in 65+ cohort	Vaccinated (% of total)	Declined-Unvaccinated (% of total)	Contraindicated/ unsuccessful (% of total)	No Records (% of total)
Age Band	65-<70	2,516,955	2,324,469 (92.35%)	46,018 (1.83%)	1,463 (0.06%)	145,005 (5.76%)
	70-<75	2,783,242	2,640,092 (94.86%)	51,107 (1.84%)	1,610 (0.06%)	90,433 (3.25%)
	75-<80	2,023,665	1,939,441 (95.84%)	34,244 (1.69%)	882 (0.04%)	49,098 (2.43%)
	80-<85	1,408,400	1,352,071 (96.00%)	27,048 (1.92%)	532 (0.04%)	28,749 (2.04%)
	85-<120	1,357,335	1,295,980 (95.48%)	29,176 (2.15%)	504 (0.04%)	31,675 (2.33%)
Sex	Female	5,445,440	5,167,911 (94.90%)	107,359 (1.97%)	2,401 (0.04%)	167,769 (3.08%)
	Male	4,644,171	4,384,163 (94.40%)	80,262 (1.73%)	2,562 (0.06%)	177,184 (3.82%)
High Level Ethnicity	White	6,384,245	6,140,638 (96.18%)	92,659 (1.45%)	2,219 (0.03%)	148,729 (2.33%)
	Mixed	40,173	32,081 (79.86%)	2,597 (6.46%)	70 (0.17%)	5,425 (13.50%)
	South Asian	310,177	270,592 (87.24%)	11,564 (3.73%)	273 (0.09%)	27,748 (8.95%)
	Black	119,616	84,245 (70.43%)	13,349 (11.16%)	175 (0.15%)	21,847 (18.26%)
	Other	62,559	48,440 (77.43%)	3,934 (6.29%)	126 (0.20%)	10,059 (16.08%)
	Unknown	3,172,813	2,976,050 (93.80%)	63,490 (2.00%)	2,135 (0.07%)	131,138 (4.13%)
IMD Band	Unknown	82,649	78,309 (94.75%)	1,617 (1.96%)	49 (0.06%)	2,674 (3.24%)
	1 (most deprived)	1,418,151	1,292,823 (91.16%)	44,093 (3.11%)	1,029 (0.07%)	80,206 (5.66%)
	2	1,750,252	1,627,276 (92.97%)	43,393 (2.48%)	840 (0.05%)	78,743 (4.50%)
	3	2,142,014	2,033,731 (94.94%)	38,437 (1.79%)	952 (0.04%)	68,894 (3.22%)
	4	2,298,051	2,203,257 (95.88%)	33,313 (1.45%)	1,029 (0.04%)	60,452 (2.63%)
	5 (least deprived)	2,398,466	2,316,650 (96.59%)	26,740 (1.11%)	1,106 (0.05%)	53,970 (2.25%)
Severe Mental Health	no	9,989,966	9,462,292 (94.72%)	183,589 (1.84%)	4,907 (0.05%)	339,178 (3.40%)
	yes	99,638	89,782 (90.11%)	4,025 (4.04%)	63 (0.06%)	5,768 (5.79%)
Learning Disability	no	10,065,412	9,529,114 (94.67%)	187,089 (1.86%)	4,956 (0.05%)	344,253 (3.42%)
	yes	24,192	22,953 (94.88%)	525 (2.17%)	21 (0.09%)	693 (2.86%)

Table S3b. CEV/At Risk. *CEV includes ages 16-69, At Risk includes ages 16-64. Pregnant/recent pregnancy includes those with a pregnancy code in the 8 months prior to 25 May.*

Category	Group	Total in CEV/At Risk cohort	Vaccinated (% of total)	Declined-Unvaccinated (% of total)	Contraindicated / unsuccessful (% of total)	No Records (% of total)
Age Band	16-<30	664,272	445,431 (67.06%)	28,014 (4.22%)	1,071 (0.16%)	189,756 (28.57%)
	30-<40	921,655	668,780 (72.56%)	34,496 (3.74%)	1,365 (0.15%)	217,014 (23.55%)
	40-<50	1,265,278	1,053,220 (83.24%)	35,203 (2.78%)	1,106 (0.09%)	175,749 (13.89%)
	50-<55	919,177	814,443 (88.61%)	22,043 (2.40%)	602 (0.07%)	82,089 (8.93%)
	55-<60	1,106,588	1,003,954 (90.73%)	23,471 (2.12%)	630 (0.06%)	78,533 (7.10%)
	60-<65	1,167,922	1,082,123 (92.65%)	22,015 (1.88%)	553 (0.05%)	63,231 (5.41%)
	65-<70	271,299	256,375 (94.50%)	5,411 (1.99%)	126 (0.05%)	9,387 (3.46%)
Sex	Female	3,139,913	2,656,472 (84.60%)	86,289 (2.75%)	2,695 (0.09%)	394,457 (12.56%)
	Male	3,176,292	2,667,875 (83.99%)	84,392 (2.66%)	2,723 (0.09%)	421,302 (13.26%)
High Level Ethnicity	White	3,553,998	3,095,582 (87.10%)	85,603 (2.41%)	2,947 (0.08%)	369,866 (10.41%)
	Mixed	89,950	62,062 (69.00%)	4,760 (5.29%)	140 (0.16%)	22,988 (25.56%)
	South Asian	572,208	459,228 (80.26%)	14,609 (2.55%)	483 (0.08%)	97,888 (17.11%)
	Black	260,589	164,234 (63.02%)	17,402 (6.68%)	308 (0.12%)	78,645 (30.18%)
	Other	90,370	64,575 (71.46%)	3,521 (3.90%)	133 (0.15%)	22,141 (24.50%)
	Unknown	1,749,083	1,478,645 (84.54%)	44,765 (2.56%)	1,428 (0.08%)	224,245 (12.82%)
IMD Band	Unknown	62,055	52,990 (85.39%)	1,498 (2.41%)	84 (0.14%)	7,483 (12.06%)
	1 (most deprived)	1,614,564	1,256,983 (77.85%)	64,393 (3.99%)	1,715 (0.11%)	291,473 (18.05%)
	2	1,396,969	1,142,288 (81.77%)	43,771 (3.13%)	1,078 (0.08%)	209,832 (15.02%)
	3	1,214,472	1,047,578 (86.26%)	28,357 (2.33%)	1,015 (0.08%)	137,522 (11.32%)
	4	1,066,555	949,151 (88.99%)	19,516 (1.83%)	903 (0.08%)	96,985 (9.09%)
	5 (least deprived)	961,576	875,350 (91.03%)	13,111 (1.36%)	665 (0.07%)	72,450 (7.53%)
Pregnant / Recent pregnancy	no	6,255,893	5,301,464 (84.74%)	167,097 (2.67%)	5,138 (0.08%)	782,194 (12.50%)
	yes	60,319	22,890 (37.95%)	3,584 (5.94%)	280 (0.46%)	33,565 (55.65%)
Severe Mental Health	no	5,912,137	5,038,740 (85.23%)	150,031 (2.54%)	4,858 (0.08%)	718,508 (12.15%)
	yes	404,068	285,614 (70.68%)	20,650 (5.11%)	553 (0.14%)	97,251 (24.07%)
Learning Disability	no	6,064,674	5,113,087 (84.31%)	160,937 (2.65%)	5,166 (0.09%)	785,484 (12.95%)
	yes	251,538	211,267 (83.99%)	9,744 (3.87%)	252 (0.10%)	30,275 (12.04%)

Table S3c. Ages 50-64. *Those with severe mental health conditions or learning disability within these age bands are, by definition, included within the CEV/At Risk group (Table S3b above).*

Category	Group	Total in 50-64 cohort	Vaccinated (% of total)	Declined-Unvaccinated (% of total)	Contraindicated / unsuccessful (% of total)	No Records (% of total)
Age Band	50-<55	3,114,629	2,605,316 (83.65%)	48,972 (1.57%)	1,855 (0.06%)	458,486 (14.72%)
	55-<60	2,804,333	2,425,836 (86.50%)	42,392 (1.51%)	1,610 (0.06%)	334,495 (11.93%)
	60-<65	2,152,038	1,911,868 (88.84%)	33,558 (1.56%)	1,267 (0.06%)	205,345 (9.54%)
Sex	Female	4,058,264	3,604,398 (88.82%)	58,457 (1.44%)	1,645 (0.04%)	393,764 (9.70%)
	Male	4,012,750	3,338,629 (83.20%)	66,465 (1.66%)	3,094 (0.08%)	604,562 (15.07%)
High Level Ethnicity	White	4,687,473	4,198,481 (89.57%)	63,665 (1.36%)	1,876 (0.04%)	423,451 (9.03%)
	Mixed	75,481	53,305 (70.62%)	2,135 (2.83%)	63 (0.08%)	19,978 (26.47%)
	South Asian	327,831	260,386 (79.43%)	5,089 (1.55%)	140 (0.04%)	62,216 (18.98%)
	Black	216,104	130,256 (60.27%)	8,043 (3.72%)	154 (0.07%)	77,651 (35.93%)
	Other	109,725	74,515 (67.91%)	2,583 (2.35%)	105 (0.10%)	32,522 (29.64%)
	Unknown	2,654,365	2,226,063 (83.86%)	43,393 (1.63%)	2,408 (0.09%)	382,501 (14.41%)
IMD Band	Unknown	69,797	61,299 (87.82%)	1,141 (1.63%)	35 (0.05%)	7,322 (10.49%)
	1 (most deprived)	1,293,838	1,005,179 (77.69%)	31,990 (2.47%)	861 (0.07%)	255,808 (19.77%)
	2	1,491,154	1,223,250 (82.03%)	29,456 (1.98%)	812 (0.05%)	237,636 (15.94%)
	3	1,665,132	1,446,137 (86.85%)	25,368 (1.52%)	903 (0.05%)	192,724 (11.57%)
	4	1,735,146	1,550,892 (89.38%)	20,776 (1.20%)	959 (0.06%)	162,519 (9.37%)
	5 (least deprived)	1,815,919	1,656,249 (91.21%)	16,177 (0.89%)	1,183 (0.07%)	142,310 (7.84%)

Figure S1. Cumulative percentage of patients recorded as declining a COVID-19 vaccination up to May 25th 2021, split by ethnicity, IMD, or age band. *Split by ethnicity for patients (a) aged 65+, (b) CEV/ At Risk, (c) aged 50-64; split by IMD for (d) aged 65+, (e) CEV/ At Risk, (f) aged 50-64; (g) split by age band for CEV / At Risk (CEV includes ages 16-69, At Risk includes ages 16-64).*

a

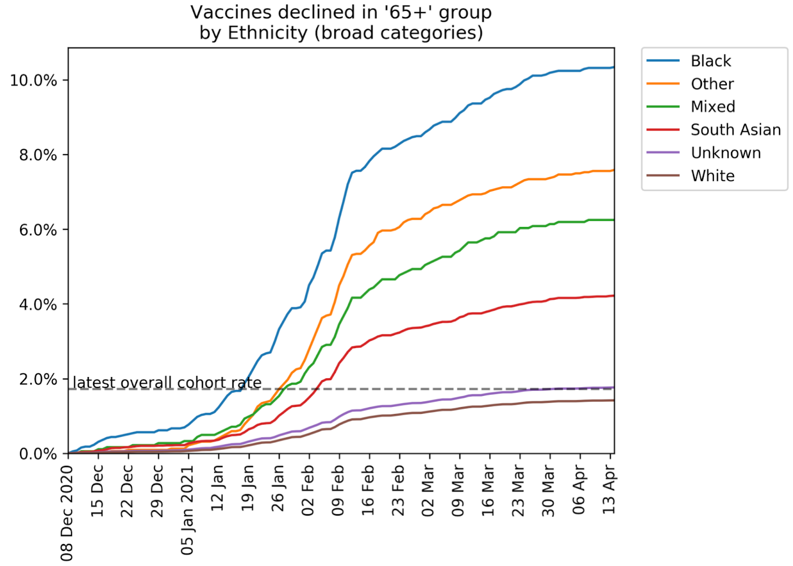

b

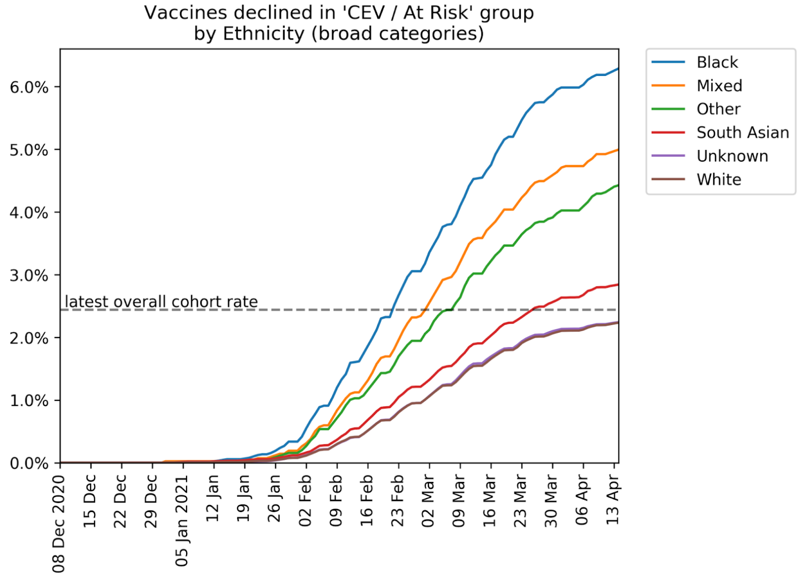

c

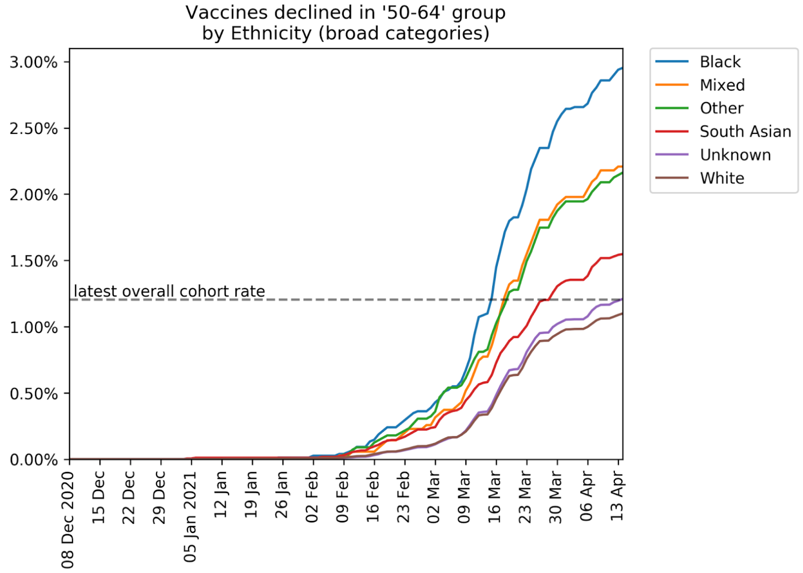
Vaccines declined in '50-64' group
by Ethnicity (broad categories)
3.00%
2.50%
2.00%
1.50%
1.00%
0.50%
0.00%
latest overall cohort rate
08 Dec 2020
15 Dec
22 Dec
29 Dec
05 Jan 2021
12 Jan
19 Jan
26 Jan
02 Feb
09 Feb
16 Feb
23 Feb
02 Mar
09 Mar
16 Mar
23 Mar
30 Mar
06 Apr
13 Apr
Black
Mixed
Other
South Asian
Unknown
White

d

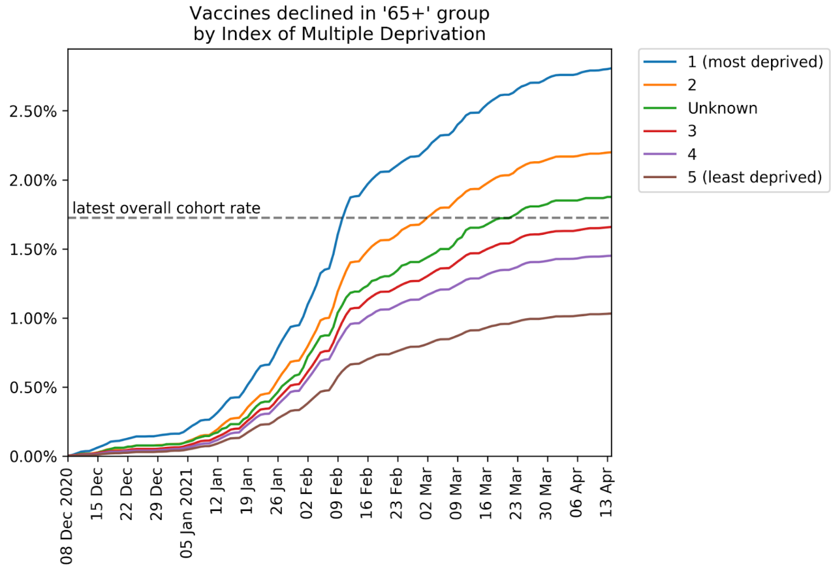
Vaccines declined in '65+' group
by Index of Multiple Deprivation
2.50%
2.00%
1.50%
1.00%
0.50%
0.00%
latest overall cohort rate
08 Dec 2020
15 Dec
22 Dec
29 Dec
05 Jan 2021
12 Jan
19 Jan
26 Jan
02 Feb
09 Feb
16 Feb
23 Feb
02 Mar
09 Mar
16 Mar
23 Mar
30 Mar
06 Apr
13 Apr
1 (most deprived)
2
Unknown
3
4
5 (least deprived)

e

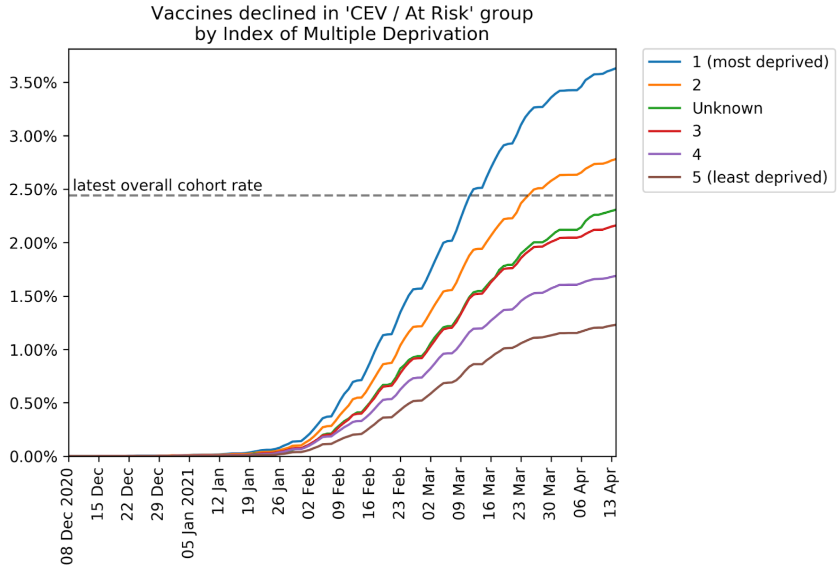
Vaccines declined in 'CEV / At Risk' group
by Index of Multiple Deprivation
3.50%
3.00%
2.50%
2.00%
1.50%
1.00%
0.50%
0.00%
latest overall cohort rate
08 Dec 2020
15 Dec
22 Dec
29 Dec
05 Jan 2021
12 Jan
19 Jan
26 Jan
02 Feb
09 Feb
16 Feb
23 Feb
02 Mar
09 Mar
16 Mar
23 Mar
30 Mar
06 Apr
13 Apr
1 (most deprived)
2
Unknown
3
4
5 (least deprived)

f

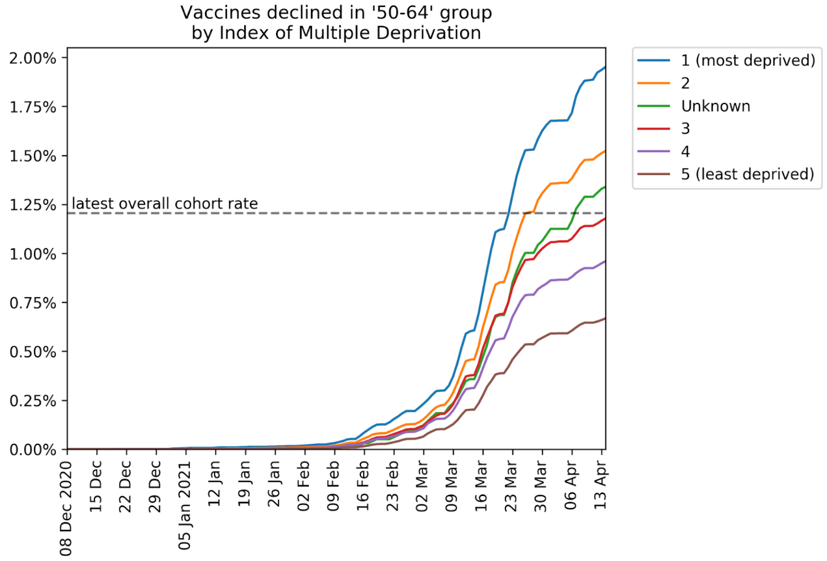
Vaccines declined in '50-64' group
by Index of Multiple Deprivation
latest overall cohort rate
2.00%
1.75%
1.50%
1.25%
1.00%
0.75%
0.50%
0.25%
0.00%
08 Dec 2020
15 Dec
22 Dec
29 Dec
05 Jan 2021
12 Jan
19 Jan
26 Jan
02 Feb
09 Feb
16 Feb
23 Feb
02 Mar
09 Mar
16 Mar
23 Mar
30 Mar
06 Apr
13 Apr
1 (most deprived)
2
Unknown
3
4
5 (least deprived)

g

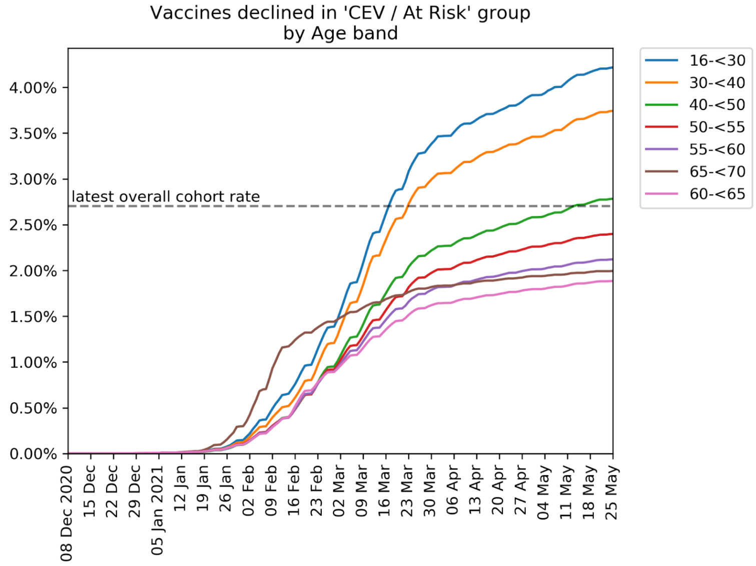
Vaccines declined in 'CEV / At Risk' group
by Age band
latest overall cohort rate
4.00%
3.50%
3.00%
2.50%
2.00%
1.50%
1.00%
0.50%
0.00%
08 Dec 2020
15 Dec
22 Dec
29 Dec
05 Jan 2021
12 Jan
19 Jan
26 Jan
02 Feb
09 Feb
16 Feb
23 Feb
02 Mar
09 Mar
16 Mar
23 Mar
30 Mar
06 Apr
13 Apr
20 Apr
27 Apr
04 May
11 May
18 May
25 May
16-<30
30-<40
40-<50
50-<55
55-<60
65-<70
60-<65

Figure S2. Heatmap showing practice variation in the number of patients recorded as declining a COVID-19 vaccination per 1000 patients in priority groups, according to the total number of priority group patients in each practice, as at 25th May 2021. *Colour scale indicates number of practices (values of 4 may represent approximated counts). Non-linear axis scales are used: the largest category on the y-axis includes all values >100. Practices with 250 or fewer registered patients in priority groups and those with 10 or fewer vaccinated patients were excluded. Patients recorded as declined only includes unvaccinated patients.*

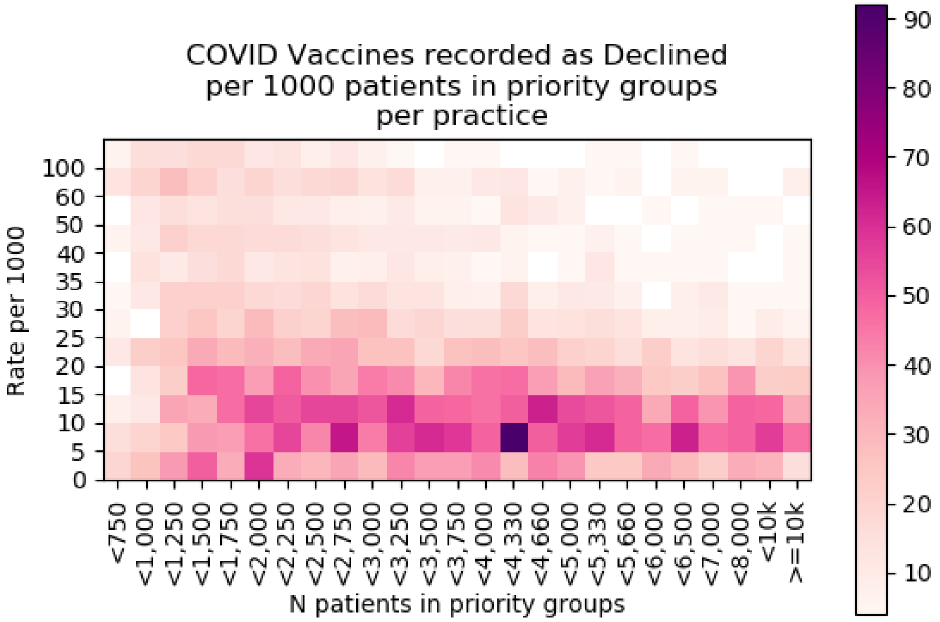

Supplement: Supplementary Material [file 2100885_SupplementaryMaterial.pdf]
